# The Synucleins and the Astrocyte

**DOI:** 10.3390/biology12020155

**Published:** 2023-01-19

**Authors:** Abigail J. Myers, Ayat Brahimi, Imani J. Jenkins, Andrew O. Koob

**Affiliations:** 1 Neuroscience Program, Health Science Research Facility, University of Vermont, 149 Beaumont Ave., Burlington, VT 05405, USA; 2 Biology Department, University of Hartford, 200 Bloomfield Ave., West Hartford, CT 06117, USA

**Keywords:** astrocyte, α-synuclein, β-synuclein, γ-synuclein, synapse, neurodegenerative disease, dementia

## Abstract

**Simple Summary:**

Emerging evidence on synucleins and astrocytes warrants closer inspection of their functional relationship. The expression and release of synucleins from the presynaptic terminal results in synuclein–astrocyte interaction. Notably, astrocytes, along with microglia, remove and degrade excess α-synuclein at the synapse. If astrocytes are impaired, toxic aggregates of α-synuclein can form in disease, and synapse loss and astrocyte dysfunction are early pathological signs of neurodegenerative disease. Less is understood about β-synuclein and γ-synuclein, although evidence indicates astrocytic uptake and expression of both proteins and possible astroprotective functions. Therefore, future research on the interconnection of synucleins and the astrocyte at the synapse will likely shed light on the mechanisms and causes of neurodegenerative disease.

**Abstract:**

Synucleins consist of three proteins exclusively expressed in vertebrates. α-Synuclein (αS) has been identified as the main proteinaceous aggregate in Lewy bodies, a pathological hallmark of many neurodegenerative diseases. Less is understood about β-synuclein (βS) and γ-synuclein (γS), although it is known βS can interact with αS in vivo to inhibit aggregation. Likewise, both γS and βS can inhibit αS’s propensity to aggregate in vitro. In the central nervous system, βS and αS, and to a lesser extent γS, are highly expressed in the neural presynaptic terminal, although they are not strictly located there, and emerging data have shown a more complex expression profile. Synapse loss and astrocyte atrophy are early aspects of degenerative diseases of the brain and correlate with disease progression. Synucleins appear to be involved in synaptic transmission, and astrocytes coordinate and organize synaptic function, with excess αS degraded by astrocytes and microglia adjacent to the synapse. βS and γS have also been observed in the astrocyte and may provide beneficial roles. The astrocytic responsibility for degradation of αS as well as emerging evidence on possible astrocytic functions of βS and γS, warrant closer inspection on astrocyte–synuclein interactions at the synapse.

## 1. Introduction

Synucleins can be expressed at various levels in skeletal muscle, cardiac muscle, the peripheral nervous system and in certain tumors, but are robustly expressed in the central nervous system (CNS) [1,2]. The distribution and extent of synuclein protein expression is dependent on tissue origin and synuclein type, as well as the condition of the surrounding cellular environment [3]. The first synuclein (α-synuclein, αS) was described as a presynaptic protein in *Torpedo californica*, localized in the electric organ [4], followed by β-synuclein (βS) as a phosphorylated 14 kDa protein in the presynaptic terminal in the rat brain [5], and then γ-synuclein (γS), which was first described in breast cancer as breast-cancer-specific gene 1 (BCSG-1) before observation in the brain and the recognition of homology with the other synucleins [6]. Subsequently, synucleins have been solely observed in vertebrates [7,8].

Astrocytes are also specific to vertebrates, as invertebrates have glial cells containing astrocyte-like function but without the same morphology [9]. Cells with true astrocyte morphology are only first observed evolutionarily in some reptiles and birds [10,11,12,13], with increasing complexity and heterogeneity in mammals and primates [9], where they are responsible for modulating most CNS functions through synaptic control [14]. Synucleins are highly expressed presynaptically, although γS resides there to a lesser extent [15,16], where they likely function to load neurotransmitter in vesicles, increase the vesicular pool and facilitate neurotransmitter release [16,17,18]. αS’s propensity to misfold and lead to toxic aggregations in neurodegenerative disease is well documented [19,20], although βS and γS aggregations have also been observed to be dysregulated in aged and/or diseased human brain [1,21], with less known about their native function. All three synucleins have also been found in glioblastomas, with αS and βS also observed in some astrocytomas [22,23].

Exosomal and soluble release of αS is internalized by astrocytes for autophagic degradation [24,25], and astrocytic accumulation of αS has been observed in neurodegenerative disease [26,27], indicating that loss of astrocytic function could result in toxic synuclein aggregations [28]. Likewise, inclusions of βS and oxidized γS have also been observed in astrocytes [29,30]. Indeed, before more cell-specific techniques, whole-brain homogenatestudies of processes such as autophagy were analyzed mainly from a neuronal perspective, without considering other cell types [31], and now it appears the astrocyte is the cell responsible for maintenance of excess synuclein protein itself and through crosstalk with microglia [32].

## 2. Synucleins and the Synapse

In humans, synucleins are mapped to separate chromosomes. The αS (aa 140) gene, *SNCA*, is mapped to chromosome 4q21.3–q22, while βS (aa 132), encoded by *SNCB*, is located on 5q35, and γS (aa 127), *SNCG*, is found on chromosome 10q23 [1]. Synucleins are small soluble proteins that consist of a highly conserved amphipathic N-terminus containing seven repeats of 11-mer with a consensus KTKEGV sequence, located between aa 7–87, with only six of the repeats in βS [1]. An acidic calcium-interacting C-terminus varies greatly between synucleins [33,34]. αS possesses a core hydrophobic NAC (non-amyloid-β component) region (aa 61–95) with a binding affinity for membranes with a small curve in diameter and folds into β-sheets that are at the core of fibrils formation in neurodegenerative diseases [35,36]. Four other human isoforms through alternative splicing of αS have been identified (aa 126, aa 112, aa 98 and aa 41); however, the native full length protein retains the most robust aggregation propensity, and other isoforms of βS and γS have yet to be identified [37,38]. βS and γS lack a NAC core, and native βS and γS aggregate less readily and rapidly than αS does [39]. Synucleins are highly expressed in the brain, and αS itself is estimated to comprise 1% of total cytosolic protein [40].

Because of their high expression and significant presence throughout the human brain, the synuclein proteins are thought to be major contributors to CNS function, specifically at the synapse. Observations in αβγ-synuclein triple knockout mice demonstrated that excitatory synapse size was decreased by nearly 30%, suggesting that synucleins support synapse structure and basic transmission [41]. In neurodegenerative diseases, synucleinopathy is common outcome, which is characterized by synapse loss and synuclein dysfunction, accumulation and release. Synucleinopathy is traditionally associated with Parkinson’s disease, dementia with Lewy bodies and multiple system atrophy. However, synuclein aggregation is observed in other diseases, and aging, as evidenced by an analysis of confirmed Alzheimer’s disease cases at the Mayo clinic, which showed that 54% also had synuclein pathology [42].

Although synuclein expression is not strictly presynaptic, most of the known function of αS and βS is due to the expression and original discovery there [15,43,44]. Synucleins can also localize in the neuronal soma and nucleus [45,46]. Likewise, the expression of γS is not as robust presynaptically as that of αS and βS, and in murine RNA-seq data, despite the higher expression in neurons, there is evidence for synuclein expression in astrocytes themselves [47,48,49,50,51]. Additionally, γS is more highly expressed in human mature astrocytes derived from iPSCs compared to neurons, as contrasted to mouse expression, where it appears to be more highly expressed in the neuron [52,53].

γS and βS can inhibit αS fibrillization, and initial in vitro studies with thioflavin T fluorescent analysis of αS while in combination with βS or γS revealed that βS and γS can inhibit the rate of αS fibrillization with a 1:1 ratio and completely abolish it at a 4:1 βS:αS or γS:αS ratio [54,55]. Additionally, γS and βS have been shown to exhibit chaperone behavior in vivo, and could inhibit protein misfolding [56,57]. Therefore, γS and βS could inhibit αS misfolding and aggregation. In αS/ βS double transgenics mice, overexpression of βS can inhibit behavioral deficits and aggregation of αS at the synapse observed in mice overexpressing αS alone [58]. Likewise, the murine neuronal expression of γS and αS at the synapse results in their ability to share at least some functional properties [59], with conflicting evidence on vesicular binding capabilities being restricted to αS function [60].

It appears that αS regulates neurotransmitter release and the transport function of synaptic vesicles, as well as maintain the size of recycling pools at synapses [18,61]. Research using WT αS, αS null and overexpressed αS cultured in mouse hippocampal neurons found that αS mitigates vesicle trafficking within synapses, effectively maintaining the number of synaptic vesicles available for release upon stimulation [62,63]. Because αS has high affinity for membranes with a small curve in diameter, it binds vesicular membranes [64,65]. Furthermore, the C-terminus of αS and cysteine-string protein-α (CSPα) support SNARE folding, which is a protein necessary for neurotransmitter release and vesicle recycling [66], and can facilitate SNARE complex formation, to promote vesicular exocytosis and transmitter release [17,67]. Because of their ability to bind to the αS region responsible for membrane binding, βS and γS can inhibit αS vesicular binding and contribution to vesicular trafficking [16]. Once αS is unbound from the vesicular membrane, it can aggregate, unless bound to βS or γS [16]. Likewise, inducing point mutations to increase βS and γS membrane affinity increases their toxicity and ability to form cytoplasmic inclusions similar to those of αS [68].

However, αS, βS and γS are not restricted to the intracellular space, as all have been observed in human cerebrospinal fluid and interstitial fluid, meaning they are constitutively released [69]. It has been shown that αS can be released via exosomes in monomeric or oligomeric form [70,71], and increased levels of synucleins are found in the cerebral spinal fluid of patients with neurodegenerative disease [54,72].

## 3. The Astrocyte and the Synapse

Astrocytes control all aspects of the synapse to promote synaptic health [73,74,75,76,77,78,79]. Their incredible diversity and malleable response and function in different brain states, from broad destructive disease and injury to micro perturbations in the healthy brain, is just beginning to be understood [80,81]. Their extensive bushy morphology contacts thousands of synapses in individual territories. They are responsible for synaptic plasticity, including synaptogenesis [82,83,84] and regulation of neurotransmission [73,85,86,87,88]. They respond to neurotransmission through discrete calcium increases in endfoot processes [89]. Intercellular transmission is not completely neuronal in the CNS, and it is known that astrocytic gliotransmission contributes to synaptic communication [14,90,91]. Subsequently, astrocytes have been shown to control neuronal network activity as a modulator of the synapse [92,93,94,95,96]. Due to this, increasing emerging evidence has shown that astrocytes orchestrate many behavioral and cognitive processes in the brain [97,98]. For example, recently, astrocyte control of anxiety and reward in the hippocampus, as well as more evidence confirming the well-established astrocytic role in learning and memory have been shown [99,100,101]. Additional recent evidence also supports the responsibility of astrocytes for affective behavior in the amygdala [102], reward in the ventral tegmental area [103], repetitive behavior and attention in the striatum [104,105], as well as modulation of sleep [106]. Lastly, more evidence reinforces astrocyte regulation of working memory in the prefrontal cortex [107,108].

Astrocytes also remove and degrade debris, damaged organelles and toxic proteinaceous accumulations at the synapse [109,110]. They can work with microglia to prune synapses through phagocytosis, with astrocytes mainly focusing on excitatory synapses [111] for circuit homeostasis. The endolysosomal pathway in astrocytes can help remove and degrade excess synaptic waste to maintain synapse integrity [109]. Damaged mitochondria in dopaminergic neurons in Parkinson’s disease are transferred to astrocytes for degradation through transmitophagy [112]. Neurons exposed to amyloid-β protofibrils will release them in exosomes which are rapidly imbibed by astrocytes [113,114]. Likewise, toxic proteins have been shown to be cleared by astrocytes during sleep via the glymphatic pathway [115,116,117,118]. Sleep deprivation also increases astrocytic phagocytic activity at the synapse [119]. Expression data comparing astrocytes in development and mature astrocytes has shown astrocytes to upregulate transcription of proteins involved in engulfment and phagocytosis until maturity [120]. Working with microglia, astrocytes are also responsible for the neuroinflammatory response in damaged or degenerative nervous tissue [121]. Proteins can be transferred from neuron to astrocyte and astrocyte to astrocyte via tunneling nanotubes, which is facilitated by the endolysosomal pathway [122,123].

Because of these responsibilities, as well as the clear evidence of an involvement in cognition, attention has turned to astrocytic dysfunction as the possible cause of neurodegenerative diseases [124,125]. Synapse loss correlates with the rate of cognitive decline in early disease states [126,127]. In conjunction with early synapse loss, astrocyte atrophy has been observed in neurodegenerative disease, including Parkinson’s disease, where an analysis of dysregulated genetic expression is also mainly astrocytic in origin [128,129]. Astrocytic dysfunction is particularly impactful to the human brain, where astrocytes in the cortex are 27 times greater in volume and have 10 times as many terminal processes, estimated to contact up to 2 × 10^6^ synapses compared with 1.2 × 10^5^ in the rodent [130,131].

## 4. α-Synuclein and Astrocytes

**Figure 1:**
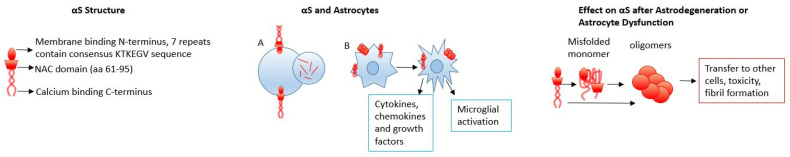
αS’s structure consists of an amphipathic membrane-binding N-terminus sequence that contains 7 repeats with the consensus KTKEGV sequence, a non-amyloid-β component (NAC) domain responsible for its aggregation potential, and a calcium binding C-terminus. In A, the astrocyte can degrade αS monomers and oligomers through the endolysosomal pathway. In B, interaction with αS monomers and oligomers can cause astrocyte reactivity resulting in the release of cytokines, chemokines and growth factors, and cause microglial activation, although the level of monomeric αS to induce broad effects is uncertain. In the event of astrodegeneration or astrocyte dysfunction, αS can misfold, aggregate and spread from cell to cell, causing toxic fibril formation, which can also then cause native αS to misfold as well.

Excess αS from neuronal presynaptic terminals [4,44] is released in soluble form or via exosomes into the extracellular space, where it is taken up by astrocytes and degraded through the endolysosmal pathway [24,25,26,27,132,133] (Figure 1A). It has recently been shown that endogenous neuronal αS does not contribute appreciably to the toxicity of αS, and that αS already aggregated from external sources interacts with mitochondria as the cause [134], placing additional focus on astrocytic function to prevent synucleinopathy.

αS can interact with mitochondria and the endolysosomal system for autophagy, and processing by the ubiqutin/proteosome pathway has been observed in neurons [135,136]. In the event of astrocyte atrophy or dysfunction—and astrocytes are unable to adequately remove and degrade αS—it can misfold and accumulate, become toxic to neurons and influence other native αS to misfold, eventually resulting in Lewy bodies [137]. Lewy bodies are abnormal inclusions largely consisting of neurofilaments, ubiquitin, and the αS protein [138,139]. The fibrillized form of αS is the most capable of inducing native αS to fibrillize [140] (Figure 1). Oligomeric αS is then the main form that can cause toxic aggregates by interacting and disrupting mitochondrial function [141]. αS oligomers leading to fibrillization and subsequent Lewy body formation cause disruption of synaptic function, which advances neurodegeneration [142].

Since cognitive impairments can present decades before histological signs, an initial decrease in astrocytic populations that coincides with early cognitive decline could also be the cause of eventual protein inclusions [143]. When the astrocytic oligomeric load increases to a point that it is causing mitochondria damage and reduced cell viability [144], αS is transferred to other healthy astrocytes via tunneling nanotubes for removal, but also facilitates the propagation of oligomeric and toxic αS [145]. Likewise, to maintain αS, a unique form of αS is observed in astrocytes due to post translational modification to remove the N and C terminus as well as phosphorylate the protein at Y39 [146].

In astrocytes derived from patient-specific induced pluripotent stem cells (iPSCs), impaired chaperone-mediated autophagy (CMA) and macroautophagy degradation of αS is observed when comparing cells from familial mutant LRRK2 G2019S and controls [147]. Increased p62, LC3-II and LAMP2 redistribution are observed in astrocytes from familial Parkinson’s disease patients, with autophagic flux less responsive to lysosomal proteolysis inhibitors [147]. Overexpression of αS and its mutant forms also decreases LC3-II expression and increases p62 expression in astrocytes, indicating impaired macroautophagy [148]. Similarly, this causes apoptosis in astrocytes, with mutant forms much more dramatic than native αS [148]. Mutations to PINK1 and Parkin, both expressed predominantly by astrocytes and essential for healthy autophagy, result in familial neurodegeneration with evidence of αS forming Lewy bodies in aged patients [149,150]

Additionally, upon internalization of αS, the genetic expression profile of astrocytes changes, with neuroinflammatory genes upregulated, resulting in initially protective astrocyte reactivity [32,151,152] that occurs along a continuum of injury or disease severity [153,154] (Figure 1B). Astrocyte reactivity can subsequently induce microglial activation [27]. This can be region-specific, as demonstrated by astrocytes in the midbrain of a mouse model of PD exhibiting a pro-inflammatory profile with macrophage/monocyte and microglia phagocytizing dopaminergic neurons, but not in the striatum, where despite a pro-inflammatory profile of microglia, neurons are not degraded [155]. αS in momomer and aggregated forms can also bind indiscriminately on various receptors to induce an inflammatory response in the microglia [156] as well as astrocytes, including TLR4 [157,158]. Astrocyte reactivity to αS has been shown in post mortem tissue of patients diagnosed with neurodegenerative disease and in tissue culture, in addition to transgenic mouse models overexpressing αS [132,144,159,160,161,162]. Growth factors, cytokines, chemokines and antioxidant enzymes are upregulated initially in astrocytes when they become reactive [163,164], and mutant glial fibrillary acidic protein (GFAP), a signature of astrocyte reactivity, in Alexander disease dysregulates autophagy [165]. Similarly, overexpression of αS in astrocytes causes increases in growth factor expression and secretion [166]. Likewise, apolipoprotein E, which is highly expressed in astrocytes and microglia as compared to neurons, is believed to be involved in astrocytic autophagy and membrane formation. The e4 allele has been linked to Alzheimer’s disease and now is believed to facilitate αS seeding and aggregation because of its deficient interaction with αS in the membrane [42].

Familial Parkinson’s disease is the result of several mutations, A53T, A30P, E46K, H50Q, A53E, G51D and T72M either in the N-terminus region or NAC core, which result in an elevated degree of aggregation, misfolding and phosphorylation as compared to those in the native form [32,33,139,167]. Overexpression of αS in astrocytes results in apoptosis with native αS, but more dramatically with A53T and A50P mutated forms [148]. Without proper astrocyte degradation of αS, it can spread cell-to-cell in a prion-like fashion [168], whereby fibril forms of αS can influence other αS proteins to aggregate and increase toxicity [20]. However, most studies that have demonstrated prion-like αS behavior have used the A53T αS form [169,170]. Therefore, although it is becoming clear that initial astrocyte dysfunction causes propagation of αS in idiopathic synucleinopathies, further studies on the native forms of αS in astrocytes instead of A53T αS need to be conducted to properly elucidate the mechanisms.

## 5. β-Synuclein and Astrocytes

Perhaps the least is known about βS and astrocytes. Preclinical AD demonstrated an increase in the cerebrospinal fluid of βS indicating that it coincides with synapse loss [171]. βS has also been observed expressed in astrocytes in culture, and βS immunoreactivity was found in astrocytes in mouse and human brain [29].

βS has a deletion of amino acid residues 53–63 in the repeat domain of the protein, as well as high C-terminal rigidity, both factors that decrease the aggregation tendency of βS [172,173]. The protein is found at high concentrations within the cytoplasm of presynaptic axon terminals, [174] and βS can inhibit αS aggregation in vivo and in vitro most effectively [175], via the C-terminus region of aa 115–134 binding to the αS N-terminus [176]. βS interaction with αS fibrils also leads to reduced seeding and toxicity [177].

**Figure 2:**
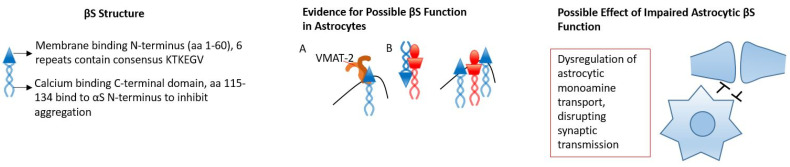
βS only contains 6 repeated sequences with the consensus of KTKEGV in the N-terminus. βS’s calcium-binding C-terminus is also responsible for the inhibition of αS aggregation by interacting with the N-terminus of αS. In A, it has been shown in neurons that βS facilitates monoamine transport through VMAT-2, which would likely result in this function in astrocytes. In B, βS inhibits detrimental αS aggregation through two methods, the C-terminus region binding with the αS N-terminus to form heterodimers and inhibit aggregation, and βS competing with αS for membrane binding. The likely effect of impaired βS functioning in astrocytes would be dysregulation of astrocytic monoamine transport from the synapse and within the astrocyte for release.

Beyond the structural support of axon terminals, βS contributes to neurological homeostasis through functions that regulate dopamine uptake, apoptosis and lipid binding [178]. Proper dopamine neurotransmission is reliant on the reuptake of dopamine into acidic synaptic vesicles via vesicular monoamine transporter-2 (VMAT-2). This reuptake is dependent on βS, as studies have shown that VMAT-2 activity significantly decreases in βS null mutant mice [178]. Intriguingly, VMAT-2 is expressed by astrocytes, and disruption to homeostatic control by VMAT-2 astrocyte knockouts causes cognitive impairments [179] (Figure 2A).

βS has also been shown to have anti-apoptotic effects. For example, neurons expressing low, physiological levels of βS are more resistant to chemically induced apoptosis as compared to mock-transfected neurons [180], and βS binding of αS has been shown to decrease αS membrane association [181].

Recent studies have also revealed a direct physiological interplay between βS and αS (Figure 2B). βS mitigates αS aggregation in a dose-dependent manner where, in conditions of equimolar βS, αS was present only in the monomeric form [182]. Additionally, βS attenuates many cytotoxic effects of αS, including the production of reactive oxygen species, inhibition of proteasomal activity and impairment of motor activity [181]. It appears βS can compete with αS binding on lipid vesicles or fibril formations in order to provide beneficial anti-aggregating effects [183]. Conversely, βS expression in rats resulted in βS aggregation and neurotoxicity, conflicting with the evidence of protective βS function [181,184]. Likewise, T-cell activation is prompted by neuronal βS in Lewis rats, a model of multiple sclerosis, which results in neurodegeneration, reactive astrocytes and activated microglia [185]. The exploration of βS expression in astrocytes in vitro or in vivo has yet to be conducted in relation to astrocyte αS processing, and the function of βS itself to further understand these processes.

Two mutants of βS (P123H and V70M) that increase aggregating properties are associated with lysosomal pathology and dementia with Lewy bodies [186,187]. The P123H mutant has been shown to induce astrocyte reactivity [188] and neuroinflammatory phenotypes in the hippocampus [189]. p123H was discovered in a familial case of DLB and is associated with the accumulation of insoluble βS, and behaviorally results in learning and memory deficits [190]. When P123H mice were crossed with αS transgenic mice, neurodegeneration worsened, further supporting the hypothesis that βS neurotoxicity may result from an imbalance in αS/βS interplay [190]. The effects of the P123H βS mutation may be due to pathological lysosomal inclusions, abnormal lipid binding and/or increased propensity for βS aggregation due to increased flexibility of the C-terminal end of the protein [181,191]. The V70M βS mutation was discovered in a case of sporadic DLB and is associated with the degeneration of both dopaminergic and non-dopaminergic neurons [192]. Unlike the P123H βS mutation, the V70M mutation has not been shown to influence neuronal network activity [192]. Additionally, when compared to native βS, both the P123H and V70M βS mutations express increased rates of fibrillation in slightly acidic microenvironments, forming structures similar to αS aggregates.

The discovery of βS in astrocytes with the beneficial inhibition of αS aggregation, and an understanding that mutations that cause aggregations of βS can cause astrocyte reactivity, indicate that studies on βS in astrocytes could be beneficial to the understanding of synucleinopathies. Likewise βS affinity for VMAT-2, a vesicular transporter also expressed by astrocytes, might indicate βS involvement in gliotransmission and astrocytic monoamine transmitter uptake at the synapse.

## 6. γ-Synuclein and Astrocytes

Initially, γS was discovered as a protein upregulated in breast cancer and named BCSG-1 [6]. In the central nervous system it was likewise observed as increased in glioblastomas [6,22,193]. γS has been shown to promote cell proliferation and radioresistance in a variety of cancer types, including glioblastoma, and is most often used as a biomarker for breast cancer diagnosis and progression [194,195]. Expression of the γS protein has also been observed in the adult rodent brain, specifically in neurons of the brainstem, thalamus, hypothalamus, hippocampus and cerebral cortex [52]. Additionally, studies have shown that human cortical astrocytes are capable of both endogenous γS expression and internalization of extracellular γS [196,197]. γS shares the least homology with other synucleins, and only 60% with αS [59].

Overexpression of mouse neuronal γS results in deficits in learning, memory and locomotor activity and causes γS inclusions in neurons and astrocytes [198,199]. Conversely, although γS knockouts result in reduced cellular proliferation in the midbrain in development, no behavioral deficits are observed [200]. Additionally, conflictingly in the rat brain, γS expression does not aggregate or appreciably cause any behavioral or degenerative effects as compared with the other synucleins [184]. However, RNA-seq data show that human neuronal γS is reduced compared to that of mice, while mature human astrocytes derived from iPSCs express higher levels of γS compared to neurons, while in mice, expression is higher in the neuron [52].

**Figure 3:**
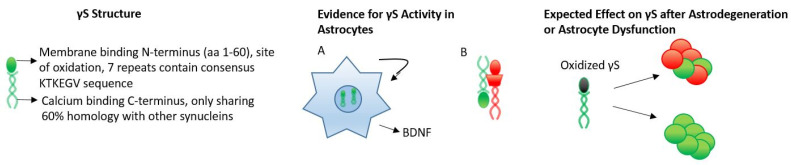
γS differs the most in sequence to the synucleins, sharing only 60% homology with αS due to its highly variable C-terminal region. Like αS, γS has 7 repeats with the KTKEGV consensus sequence in the N-terminal region. In A, it has been shown that γS is capable of inducing astrogenesis and astroprotection through increased expression and release of BDNF. Similarly, knockdown of γS in astrocytes results in a mitotic catastrophe and apoptosis. In B, it has been shown that γS can inhibit αS aggregation, which has not been studied in vivo, and could possibly occur in astrocytes, where it is expressed. Oxidation of γS results in toxic aggregations of γS itself, influencing the aggregation of αS. As reactive oxygen species’ production is a hallmark of astrocyte dysfunction or astrodegeneration, detrimental oxidized γS would likely be a byproduct.

It has been shown in human astrocytes in tissue culture that γS may be astroprotective. When human astrocytes in tissue culture are treated with physiological levels of extracellular γS, it is internalized and stimulates cellular proliferation, which is followed by increased cell viability and expression and release of neuroprotective brain-derived neurotrophic factor (BDNF) [196]. Likewise, RNAi knockdown of endogenous human astrocytic γS reduces cellular proliferation, increases apoptosis and upregulates phospho-histone H3 to indicate arrest with chromosome condensation and subsequent cell death [197] (Figure 3A).

In neurons, in neurodegenerative disease, cell cycle arrest and mitotic catastrophe have been shown [22,23,30,58,69,201,202], therefore, astrocytic γS dysregulation could have adverse effects intercellularly by inducing abnormal cell cycle re-entry [203]. In the astrocyte, γS could beneficially allow successful cell cycle re-entry in vivo in the adult CNS [204,205]. Studies in cancer have shown that γS regulates cell division through interaction with BuBR1, a mitotic spindle protein, which causes BuBR1 degradation and facilitates the cell to pass through the M-phase [23,206]. In addition to protein degradation of BubR1, γS also interferes with BubR1/centromere protein E interaction in checkpoint signaling, and through ERK 1/2 stimulates protective MAP kinase pathways [207]. An exploration of this mechanism in primary astrocytes could provide a window into the native function of γS.

Similarly, age-associated glaucoma and optic nerve degeneration correlates with reduced γS expression, further implicating protective γS properties in the nervous system [208]. It has also been shown that γS can inhibit αS fibrillization in vitro, indicating possible protective properties in neurodegeneration [54,55] (Figure 3B). However, oxidized γS is capable of aggregation, and overexpression can lead to the death of motor neurons, impaired synaptic vesicle release and synaptic dysfunction [198,209]. This aggregation of γS results from oxidization of the Met^38^ and Tyr^39^ residues in the synuclein, which has also been found to promote αS misfolding, aggregation and toxicity [210] (Figure 3). However, oxidized γS and its effects have yet to be explored in the astrocyte. Similarly, γS coincides with αS in human pathological lesions in the brain and is increased in the CSF of Alzheimer’s disease patients [30]. The increased presence of γS has also been noted within the cerebrospinal fluid of patients with Alzheimer’s disease and dementia with Lewy bodies [22,69].

Lastly, synaptic dysregulation is a hallmark of autism spectrum disorder (ASD), with astrocytic dysfunction being considered as a possible cause [211,212]. In ASD, plasma levels of γS are significantly decreased, while αS is increased [213]. αS/γS antagonism is only moderately studied, specifically in relation to neurological diseases that involve damaging protein aggregations. Information about both their independent and combined effects could promote further understanding of the synucleinopathies, leading to better outcomes for those diagnosed with neurodegenerative disease.

## 7. Discussion

More research on how βS and γS affect αS in the astrocyte would provide beneficial knowledge on synuclein function and the cause and treatment of synucleinopathies. Astrocytes promote synaptogenesis, synaptic health, contribute to synaptic communication [73] and remove neuronally derived αS from the extracellular space [25]. Both synapse loss and astrocyte atrophy are prevalent in the aged brain and in early stages of neurodegenerative disease [126,128,214]. αS accumulation, toxicity and prion-like propagation in humans could be a consequence of initial astrocytic cell death or dysfunction.

Both βS and γS can inhibit αS fibrillization and have both been shown to be protective. The therapeutic benefits of this are unclear, as oxidized γS can be toxic and facilitate αS aggregation [210], whereas some conflicting studies indicate that βS can also be toxic and may compete with αS function at the vesicle, which could result in subsequent αS aggregation. However, as astrocytes are responsible for the degradation of αS, and astrocyte dysfunction would result in synucleinopathy, studies on γS and βS along with αS in the astrocyte would illuminate the mechanisms behind the tergiversation. Additionally, from a physiological perspective, the emerging evidence of synuclein expression by the astrocyte [47,48,49,50,51,52,53] may indicate synuclein involvement in gliotransmission or transmitter uptake, something that has not been explored. The effects of altering βS and γS expression in astrocytes would provide insight into their function and their relationship to astrocytic αS interaction.

Therefore, due to γS’s astrocytic expression in human cells and astroprotective effects, as well as the emerging evidence on βS expression, VMAT2 activity, chaperone ability and ability to inhibit αS aggregation, further exploration on the role of synucleins is warranted, particularly for γS and βS on astrocytic function, gliotransmission and endolysosomal processing of αS.

## Data Availability

No new data were created or analyzed in this study. Data sharing is not applicable to this article.

## References

[1] George J.M. (2002). The Synucleins. Genome Biol..

[2] Lavedan C. (1998). The Synuclein Family. Genome Res..

[3] Maroteaux L., Scheller R.H. (1991). The Rat Brain Synucleins; Family of Proteins Transiently Associated with Neuronal Membrane. Brain Res. Mol. Brain Res..

[4] Maroteaux L., Campanelli J.T., Scheller R.H. (1988). Synuclein: A Neuron-Specific Protein Localized to the Nucleus and Presynaptic Nerve Terminal. J. Neurosci..

[5] Nakajo S., Tsukada K., Omata K., Nakamura Y., Nakaya K. (1993). A New Brain-Specific 14-KDa Protein Is a Phosphoprotein. Its Complete Amino Acid Sequence and Evidence for Phosphorylation. Eur. J. Biochem..

[6] Ji H., Liu Y.E., Jia T., Wang M., Liu J., Xiao G., Joseph B.K., Rosen C., Shi Y.E. (1997). Identification of a Breast Cancer-Specific Gene, BCSG1, by Direct Differential CDNA Sequencing. Cancer Res..

[7] Bonaccorsi di Patti M.C., Angiulli E., Casini A., Vaccaro R., Cioni C., Toni M. (2022). Synuclein Analysis in Adult Xenopus Laevis. Int. J. Mol. Sci..

[8] Toni M., Cioni C. (2015). Fish Synucleins: An Update. Mar. Drugs.

[9] Falcone C. (2022). Evolution of Astrocytes: From Invertebrates to Vertebrates. Front. cell Dev. Biol..

[10] Kálmán M., Pritz M.B. (2001). Glial Fibrillary Acidic Protein-Immunopositive Structures in the Brain of a Crocodilian, Caiman Crocodilus, and Its Bearing on the Evolution of Astroglia. J. Comp. Neurol..

[11] Bodega G., Suárez I., Rubio M., Fernández B. (1990). Distribution and Characteristics of the Different Astroglial Cell Types in the Adult Lizard (Lacerta Lepida) Spinal Cord. Anat. Embryol..

[12] Kovacs G.G., Breydo L., Green R., Kis V., Puska G., Lorincz P., Perju-Dumbrava L., Giera R., Pirker W., Lutz M. (2014). Intracellular Processing of Disease-Associated Alpha-Synuclein in the Human Brain Suggests Prion-like Cell-to-Cell Spread. Neurobiol. Dis..

[13] King J.S. (1966). A Comparative Investigation of Neuroglia in Representative Vertebrates: A Silver Carbonate Study. J. Morphol..

[14] Araque A., Carmignoto G., Haydon P.G., Oliet S.H.R., Robitaille R., Volterra A. (2014). Gliotransmitters Travel in Time and Space. Neuron.

[15] Iwai A., Masliah E., Yoshimoto M., Ge N., Flanagan L., Rohan de Silva H., Kittel A., Saitoh T. (1995). The Precursor Protein of Non-Aβ Component of Alzheimer’s Disease Amyloid Is a Presynaptic Protein of the Central Nervous System. Neuron.

[16] Carnazza K.E., Komer L.E., Xie Y.X., Pineda A., Briano J.A., Gao V., Na Y., Ramlall T., Buchman V.L., Eliezer D. (2022). Synaptic Vesicle Binding of α-Synuclein Is Modulated by β- and γ-Synucleins. Cell Rep..

[17] Burre J., Sharma M., Tsetsenis T., Buchman V., Etherton M.R., Sudhof T.C. (2010). α-Synuclein Promotes SNARE-Complex Assembly in Vivo and in Vitro. Science.

[18] Murphy D.D., Rueter S.M., Trojanowski J.Q., Lee V.M. (2000). Synucleins Are Developmentally Expressed, and Alpha-Synuclein Regulates the Size of the Presynaptic Vesicular Pool in Primary Hippocampal Neurons. J. Neurosci. Off. J. Soc. Neurosci..

[19] Dettmer U., Newman A.J., von Saucken V.E., Bartels T., Selkoe D. (2015). KTKEGV Repeat Motifs Are Key Mediators of Normal α-Synuclein Tetramerization: Their Mutation Causes Excess Monomers and Neurotoxicity. Proc. Natl. Acad. Sci..

[20] Goedert M., Masuda-Suzukake M., Falcon B. (2016). Like Prions: The Propagation of Aggregated Tau and Alpha-Synuclein in Neurodegeneration. Brain.

[21] Goedert M., Jakes R., Spillantini M.G. (2017). The Synucleinopathies: Twenty Years On. J. Parkinsons. Dis..

[22] Fung K.-M., Rorke L.B., Giasson B., Lee V.M.-Y., Trojanowski J.Q. (2003). Expression of Alpha-, Beta-, and Gamma-Synuclein in Glial Tumors and Medulloblastomas. Acta Neuropathol..

[23] Ahmad M., Attoub S., Singh M.N., Martin F.L., El-Agnaf O.M.A. (2007). Gamma-Synuclein and the Progression of Cancer. FASEB J. Off. Publ. Fed. Am. Soc. Exp. Biol..

[24] Ngolab J., Trinh I., Rockenstein E., Mante M., Florio J., Trejo M., Masliah D., Adame A., Masliah E., Rissman R.A. (2017). Brain-Derived Exosomes from Dementia with Lewy Bodies Propagate Alpha-Synuclein Pathology. Acta Neuropathol. Commun..

[25] Loria F., Vargas J.Y., Bousset L., Syan S., Salles A., Melki R., Zurzolo C. (2017). Alpha-Synuclein Transfer between Neurons and Astrocytes Indicates That Astrocytes Play a Role in Degradation Rather than in Spreading. Acta Neuropathol..

[26] Wakabayashi K., Hayashi S., Yoshimoto M., Kudo H., Takahashi H. (2000). NACP/Alpha-Synuclein-Positive Filamentous Inclusions in Astrocytes and Oligodendrocytes of Parkinson’s Disease Brains. Acta Neuropathol..

[27] Lee H., Suk J., Patrick C., Bae E., Chio J., Rho S., Hwang D., Masliah E., Lee S. (2010). Direct Transfer of Alpha-Synucelin from Neuron to Astroglia Causes Inflammatory Responses in Synucleinopathies. J Biol Chem.

[28] Yang Y., Song J.-J., Choi Y.R., Kim S.-H., Seok M.-J., Wulansari N., Darsono W.H.W., Kwon O.-C., Chang M.-Y., Park S.M. (2022). Therapeutic Functions of Astrocytes to Treat α-Synuclein Pathology in Parkinson’s Disease. Proc. Natl. Acad. Sci. USA.

[29] Tanji K., Mori F., Nakajo S., Imaizumi T., Yoshida H., Hirabayashi T., Yoshimoto M., Satoh K., Takahashi H., Wakabayashi K. (2001). Expression of Beta-Synuclein in Normal Human Astrocytes. Neuroreport.

[30] Surgucheva I., Newell K.L., Burns J., Surguchov A. (2013). New α- and γ-Synuclein Immunopathological Lesions in Human Brain. Acta Neuropathol. Commun..

[31] Croisier E., Graeber M.B. (2006). Glial Degeneration and Reactive Gliosis in Alpha-Synucleinopathies: The Emerging Concept of Primary Gliodegeneration. Acta Neuropathol..

[32] Sorrentino Z.A., Giasson B.I., Chakrabarty P. (2019). α-Synuclein and Astrocytes: Tracing the Pathways from Homeostasis to Neurodegeneration in Lewy Body Disease. Acta Neuropathol..

[33] Surguchov A., Surguchev A. (2022). Synucleins: New Data on Misfolding, Aggregation and Role in Diseases. Biomedicines.

[34] Venda L.L., Cragg S.J., Buchman V.L., Wade-Martins R. (2010). α-Synuclein and Dopamine at the Crossroads of Parkinson’s Disease. Trends Neurosci..

[35] Rodriguez J.A., Ivanova M.I., Sawaya M.R., Cascio D., Reyes F.E., Shi D., Sangwan S., Guenther E.L., Johnson L.M., Zhang M. (2015). Structure of the Toxic Core of α-Synuclein from Invisible Crystals. Nature.

[36] Qin Z., Hu D., Han S., Hong D.P., Fink A.L. (2007). Role of different regions of α-synuclein in the assembly of fibrils. Biochemistry.

[37] Bungeroth M., Appenzeller S., Regulin A., Völker W., Lorenzen I., Grötzinger J., Pendziwiat M., Kuhlenbäumer G. (2014). Differential Aggregation Properties of Alpha-Synuclein Isoforms. Neurobiol. Aging.

[38] Vinnakota R.L., Yedlapudi D., Manda K.M., Bhamidipati K., Bommakanti K.T., RangaLakshmi G.S., Kalivendi S. (2018). V Identification of an Alternatively Spliced α-Synuclein Isoform That Generates a 41-Amino Acid N-Terminal Truncated Peptide, 41-Syn: Role in Dopamine Homeostasis. ACS Chem. Neurosci..

[39] Biere A.L., Wood S.J., Wypych J., Steavenson S., Jiang Y., Anafi D., Jacobsen F.W., Jarosinski M.A., Wu G.M., Louis J.C. (2000). Parkinson’s Disease-Associated Alpha-Synuclein Is More Fibrillogenic than Beta- and Gamma-Synuclein and Cannot Cross-Seed Its Homologs. J. Biol. Chem..

[40] Stefanis L. (2012). α-Synuclein in Parkinson’s Disease. Cold Spring Harb. Perspect. Med..

[41] Greten-Harrison B., Polydoro M., Morimoto-Tomita M., Diao L., Williams A.M., Nie E.H., Makani S., Tian N., Castillo P.E., Buchman V.L. (2010). Aβγ-Synuclein Triple Knockout Mice Revealage-Dependent Neuronal Dysfunction. Proc. Natl. Acad. Sci. USA.

[42] Jin Y., Li F., Sonoustoun B., Kondru N.C., Martens Y.A., Qiao W., Heckman M.G., Ikezu T.C., Li Z., Burgess J.D. (2022). APOE4 Exacerbates α-Synuclein Seeding Activity and Contributes to Neurotoxicity in Alzheimer’s Disease with Lewy Body Pathology. Acta Neuropathol..

[43] Specht C.G., Tigaret C.M., Rast G.F., Thalhammer A., Rudhard Y., Schoepfer R. (2005). Subcellular Localisation of Recombinant Alpha- and Gamma-Synuclein. Mol. Cell. Neurosci..

[44] Spinelli K.J., Taylor J.K., Osterberg V.R., Churchill M.J., Pollock E., Moore C., Meshul C.K., Unni V.K. (2014). Presynaptic Alpha-Synuclein Aggregation in a Mouse Model of Parkinson’s Disease. J. Neurosci..

[45] Mori F., Tanji K., Yoshimoto M., Takahashi H., Wakabayashi K. (2002). Immunohistochemical Comparison of Alpha- and Beta-Synuclein in Adult Rat Central Nervous System. Brain Res..

[46] Surgucheva I., McMahon B., Surguchov A. (2006). Gamma-Synuclein Has a Dynamic Intracellular Localization. Cell Motil. Cytoskeleton.

[47] Clarke L.E., Liddelow S.A., Chakraborty C., Münch A.E., Heiman M., Barres B.A. (2018). Normal Aging Induces A1-like Astrocyte Reactivity. Proc. Natl. Acad. Sci. USA.

[48] Yu X., Nagai J., Marti-Solano M., Soto J.S., Coppola G., Babu M.M., Khakh B.S. (2020). Context-Specific Striatal Astrocyte Molecular Responses Are Phenotypically Exploitable. Neuron.

[49] Srinivasan R., Lu T.-Y., Chai H., Xu J., Huang B.S., Golshani P., Coppola G., Khakh B.S. (2016). New Transgenic Mouse Lines for Selectively Targeting Astrocytes and Studying Calcium Signals in Astrocyte Processes In Situ and In Vivo. Neuron.

[50] Chai H., Diaz-Castro B., Shigetomi E., Monte E., Octeau J.C., Yu X., Cohn W., Rajendran P.S., Vondriska T.M., Whitelegge J.P. (2017). Neural Circuit-Specialized Astrocytes: Transcriptomic, Proteomic, Morphological, and Functional Evidence. Neuron.

[51] Diaz-Castro B., Bernstein A.M., Coppola G., Sofroniew M.V., Khakh B.S. (2021). Molecular and Functional Properties of Cortical Astrocytes during Peripherally Induced Neuroinflammation. Cell Rep..

[52] Zhang Y., Sloan S.A., Clarke L.E., Caneda C., Plaza C.A., Blumenthal P.D., Vogel H., Steinberg G.K., Edwards M.S.B., Li G. (2016). Purification and Characterization of Progenitor and Mature Human Astrocytes Reveals Transcriptional and Functional Differences with Mouse. Neuron.

[53] Zhang Y., Chen K., Sloan S.A., Bennett M.L., Scholze A.R., O’Keeffe S., Phatnani H.P., Guarnieri P., Caneda C., Ruderisch N. (2014). An RNA-Sequencing Transcriptome and Splicing Database of Glia, Neurons, and Vascular Cells of the Cerebral Cortex. J. Neurosci. Off. J. Soc. Neurosci..

[54] Uversky V.N., Li J., Souillac P., Millett I.S., Doniach S., Jakes R., Goedert M., Fink A.L. (2002). Biophysical Properties of the Synucleins and Their Propensities to Fibrillate. J. Biol. Chem..

[55] Sanjeev A., Mattaparthi V.S.K. (2019). Computational Study on the Role of Gamma-Synuclein in Inhibiting the Alpha-Synuclein Aggregation. Cent. Nerv. Syst. Agents Med. Chem..

[56] Jiang Y., Liu Y.E., Goldberg I.D., Shi Y.E. (2004). Gamma Synuclein, a Novel Heat-Shock Protein-Associated Chaperone, Stimulates Ligand-Dependent Estrogen Receptor Alpha Signaling and Mammary Tumorigenesis. Cancer Res..

[57] Surgucheva I., Ninkina N., Buchman V.L., Grasing K., Surguchov A. (2005). Protein Aggregation in Retinal Cells and Approaches to Cell Protection. Cell. Mol. Neurobiol..

[58] Hashimoto M., Rockenstein E., Mante M., Mallory M., Masliah E. (2001). Beta-Synuclein Inhibits Alpha-Synuclein Aggregation: A Possible Role as an Anti-Parkinsonian Factor. Neuron.

[59] Kuhn M., Haebig K., Bonin M., Ninkina N., Buchman V.L., Poths S., Riess O. (2007). Whole Genome Expression Analyses of Single- and Double-Knock-out Mice Implicate Partially Overlapping Functions of Alpha- and Gamma-Synuclein. Neurogenetics.

[60] Ninkina N., Peters O.M., Connor-Robson N., Lytkina O., Sharfeddin E., Buchman V.L. (2012). Contrasting Effects of Alpha-Synuclein and Gamma-Synuclein on the Phenotype of Cysteine String Protein Alpha (CSPalpha) Null Mutant Mice Suggest Distinct Function of These Proteins in Neuronal Synapses. J. Biol. Chem..

[61] Cabin D.E., Shimazu K., Murphy D., Cole N.B., Gottschalk W., McIlwain K.L., Orrison B., Chen A., Ellis C.E., Paylor R. (2002). Synaptic Vesicle Depletion Correlates with Attenuated Synaptic Responses to Prolonged Repetitive Stimulation in Mice Lacking Alpha-Synuclein. J. Neurosci. Off. J. Soc. Neurosci..

[62] Scott D., Roy S. (2012). α-Synuclein Inhibits Intersynaptic Vesicle Mobility and Maintains Recycling-Pool Homeostasis. J. Neurosci..

[63] Denker A., Rizzoli S.O. (2010). Synaptic Vesicle Pools: An Update. Front. Synaptic Neurosci..

[64] Davidson W.S., Jonas A., Clayton D.F., George J.M. (1998). Stabilization of Alpha-Synuclein Secondary Structure upon Binding to Synthetic Membranes. J. Biol. Chem..

[65] Middleton E.R., Rhoades E. (2010). Effects of Curvature and Composition on α-Synuclein Binding to Lipid Vesicles. Biophys. J..

[66] Chandra S., Gallardo G., Fernández-Chacón R., Schlüter O.M., Südhof T.C. (2005). α-Synuclein Cooperates with CSPα in Preventing Neurodegeneration. Cell.

[67] Burré J., Sharma M., Südhof T.C. (2014). α-Synuclein Assembles into Higher-Order Multimers upon Membrane Binding to Promote SNARE Complex Formation. Proc. Natl. Acad. Sci. USA.

[68] Kim T.-E., Newman A.J., Imberdis T., Brontesi L., Tripathi A., Ramalingam N., Fanning S., Selkoe D., Dettmer U. (2021). Excess Membrane Binding of Monomeric Alpha-, Beta- and Gamma-Synuclein Is Invariably Associated with Inclusion Formation and Toxicity. Hum. Mol. Genet..

[69] Oeckl P., Metzger F., Nagl M., von Arnim C.A.F., Halbgebauer S., Steinacker P., Ludolph A.C., Otto M. (2016). Alpha-, Beta-, and Gamma-Synuclein Quantification in Cerebrospinal Fluid by Multiple Reaction Monitoring Reveals Increased Concentrations in Alzheimer′s and Creutzfeldt-Jakob Disease but No Alteration in Synucleinopathies. Mol. Cell. Proteomics.

[70] Danzer K.M., Kranich L.R., Ruf W.P., Cagsal-Getkin O., Winslow A.R., Zhu L., Vanderburg C.R., McLean P.J. (2012). Exosomal Cell-to-Cell Transmission of Alpha Synuclein Oligomers. Mol. Neurodegener..

[71] Emmanouilidou E., Melachroinou K., Roumeliotis T., Garbis S.D., Ntzouni M., Margaritis L.H., Stefanis L., Vekrellis K. (2010). Cell-Produced Alpha-Synuclein Is Secreted in a Calcium-Dependent Manner by Exosomes and Impacts Neuronal Survival. J. Neurosci. Off. J. Soc. Neurosci..

[72] Mukaetova-Ladinska E.B., Milne J., Andras A., Abdel-All Z., Cerejeira J., Greally E., Robson J., Jaros E., Perry R., McKeith I.G. (2008). Alpha- and Gamma-Synuclein Proteins Are Present in Cerebrospinal Fluid and Are Increased in Aged Subjects with Neurodegenerative and Vascular Changes. Dement Geriatr Cogn Disord.

[73] Araque A., Parpura V., Sanzgiri R.P., Haydon P.G. (1999). Tripartite Synapses: Glia, the Unacknowledged Partner. Trends Neurosci.

[74] Verkhratsky A., Nedergaard M. (2014). Astroglial Cradle in the Life of the Synapse. Philos Trans R Soc L. B Biol Sci.

[75] Haydon P.G. (2001). Glia: Listening and Talking to the Synapse. Nat. Rev. Neurosci..

[76] Christopherson K.S., Ullian E.M., Stokes C.C.A., Mullowney C.E., Hell J.W., Agah A., Lawler J., Mosher D.F., Bornstein P., Barres B.A. (2005). Thrombospondins Are Astrocyte-Secreted Proteins That Promote CNS Synaptogenesis. Cell.

[77] Ullian E.M., Sapperstein S.K., Christopherson K.S., Barres B.A. (2001). Control of Synapse Number by Glia. Science.

[78] Mauch D.H., Nagler K., Schumacher S., Goritz C., Muller E.C., Otto A., Pfrieger F.W. (2001). CNS Synaptogenesis Promoted by Glia-Derived Cholesterol. Science.

[79] Papouin T., Dunphy J., Tolman M., Foley J.C., Haydon P.G. (2017). Astrocytic Control of Synaptic Function. Philos. Trans. R. Soc. Lond. B. Biol. Sci..

[80] Endo F., Kasai A., Soto J.S., Yu X., Qu Z., Hashimoto H., Gradinaru V., Kawaguchi R., Khakh B.S. (2022). Molecular Basis of Astrocyte Diversity and Morphology across the CNS in Health and Disease. Science.

[81] Leng K., Rose I.V.L., Kim H., Xia W., Romero-Fernandez W., Rooney B., Koontz M., Li E., Ao Y., Wang S. (2022). CRISPRi Screens in Human IPSC-Derived Astrocytes Elucidate Regulators of Distinct Inflammatory Reactive States. Nat. Neurosci..

[82] Stogsdill J.A., Ramirez J., Liu D., Kim Y.H., Baldwin K.T., Enustun E., Ejikeme T., Ji R.-R., Eroglu C. (2017). Astrocytic Neuroligins Control Astrocyte Morphogenesis and Synaptogenesis. Nature.

[83] Allen N.J., Bennett M.L., Foo L.C., Wang G.X., Chakraborty C., Smith S.J., Barres B.A. (2012). Astrocyte Glypicans 4 and 6 Promote Formation of Excitatory Synapses via GluA1 AMPA Receptors. Nature.

[84] Stellwagen D., Malenka R.C. (2006). Synaptic Scaling Mediated by Glial TNF-Alpha. Nature.

[85] Sancho L., Contreras M., Allen N.J. (2021). Glia as Sculptors of Synaptic Plasticity. Neurosci. Res..

[86] Panatier A., Vallée J., Haber M., Murai K.K., Lacaille J.-C., Robitaille R. (2011). Astrocytes Are Endogenous Regulators of Basal Transmission at Central Synapses. Cell.

[87] Matos M., Bosson A., Riebe I., Reynell C., Vallée J., Laplante I., Panatier A., Robitaille R., Lacaille J.-C. (2018). Astrocytes Detect and Upregulate Transmission at Inhibitory Synapses of Somatostatin Interneurons onto Pyramidal Cells. Nat. Commun..

[88] Di Castro M.A., Volterra A. (2021). Astrocyte Control of the Entorhinal Cortex-Dentate Gyrus Circuit: Relevance to Cognitive Processing and Impairment in Pathology. Glia.

[89] Denizot A., Arizono M., Nägerl U.V., Berry H., De Schutter E. (2022). Control of Ca(2+) Signals by Astrocyte Nanoscale Morphology at Tripartite Synapses. Glia.

[90] Fellin T., Halassa M.M., Terunuma M., Succol F., Takano H., Frank M., Moss S.J., Haydon P.G. (2009). Endogenous Nonneuronal Modulators of Synaptic Transmission Control Cortical Slow Oscillations in Vivo. Proc. Natl. Acad. Sci. USA.

[91] Petrelli F., Bezzi P. (2016). Novel Insights into Gliotransmitters. Curr. Opin. Pharmacol..

[92] Poskanzer K.E., Yuste R. (2011). Astrocytic Regulation of Cortical UP States. Proc. Natl. Acad. Sci. USA.

[93] Kastanenka K.V., Moreno-Bote R., De Pittà M., Perea G., Eraso-Pichot A., Masgrau R., Poskanzer K.E., Galea E. (2020). A Roadmap to Integrate Astrocytes into Systems Neuroscience. Glia.

[94] Chever O., Dossi E., Pannasch U., Derangeon M., Rouach N. (2016). Astroglial Networks Promote Neuronal Coordination. Sci. Signal..

[95] Vasile F., Dossi E., Moulard J., Ezan P., Lecoin L., Cohen-Salmon M., Mailly P., Le Bert M., Couillin I., Bemelmans A. (2022). Pannexin 1 Activity in Astroglia Sets Hippocampal Neuronal Network Patterns. PLoS Biol..

[96] Nagai J., Yu X., Papouin T., Cheong E., Freeman M.R., Monk K.R., Hastings M.H., Haydon P.G., Rowitch D., Shaham S. (2021). Behaviorally Consequential Astrocytic Regulation of Neural Circuits. Neuron.

[97] Koob A.O. (2022). Astrocytes Imagined. J. Integr. Neurosci..

[98] Kofuji P., Araque A. (2021). Astrocytes and Behavior. Annu. Rev. Neurosci..

[99] Kol A., Adamsky A., Groysman M., Kreisel T., London M., Goshen I. (2020). Astrocytes Contribute to Remote Memory Formation by Modulating Hippocampal-Cortical Communication during Learning. Nat. Neurosci..

[100] Cho W.-H., Noh K., Lee B.H., Barcelon E., Jun S.B., Park H.Y., Lee S.J. (2022). Hippocampal Astrocytes Modulate Anxiety-like Behavior. Nat. Commun..

[101] Doron A., Rubin A., Benmelech-Chovav A., Benaim N., Carmi T., Refaeli R., Novick N., Kreisel T., Ziv Y., Goshen I. (2022). Hippocampal Astrocytes Encode Reward Location. Nature.

[102] Wahis J., Baudon A., Althammer F., Kerspern D., Goyon S., Hagiwara D., Lefevre A., Barteczko L., Boury-Jamot B., Bellanger B. (2021). Astrocytes Mediate the Effect of Oxytocin in the Central Amygdala on Neuronal Activity and Affective States in Rodents. Nat. Neurosci..

[103] Requie L.M., Gómez-Gonzalo M., Speggiorin M., Managò F., Melone M., Congiu M., Chiavegato A., Lia A., Zonta M., Losi G. (2022). Astrocytes Mediate Long-Lasting Synaptic Regulation of Ventral Tegmental Area Dopamine Neurons. Nat. Neurosci..

[104] Yu X., Taylor A.M.W., Nagai J., Golshani P., Evans C.J., Coppola G., Khakh B.S. (2018). Reducing Astrocyte Calcium Signaling In Vivo Alters Striatal Microcircuits and Causes Repetitive Behavior. Neuron.

[105] Nagai J., Rajbhandari A.K., Gangwani M.R., Hachisuka A., Coppola G., Masmanidis S.C., Fanselow M.S., Khakh B.S. (2019). Hyperactivity with Disrupted Attention by Activation of an Astrocyte Synaptogenic Cue. Cell.

[106] Vaidyanathan T.V., Collard M., Yokoyama S., Reitman M.E., Poskanzer K.E. (2021). Cortical Astrocytes Independently Regulate Sleep Depth and Duration via Separate GPCR Pathways. Elife.

[107] Tao X.-D., Liu Z.-R., Zhang Y.-Q., Zhang X.-H. (2021). Connexin43 Hemichannels Contribute to Working Memory and Excitatory Synaptic Transmission of Pyramidal Neurons in the Prefrontal Cortex of Rats. Life Sci..

[108] Mederos S., Sánchez-Puelles C., Esparza J., Valero M., Ponomarenko A., Perea G. (2021). GABAergic Signaling to Astrocytes in the Prefrontal Cortex Sustains Goal-Directed Behaviors. Nat. Neurosci..

[109] Morales I., Sanchez A., Rodriguez-Sabate C., Rodriguez M. (2017). Striatal Astrocytes Engulf Dopaminergic Debris in Parkinson’s Disease: A Study in an Animal Model. PLoS One.

[110] Tremblay M.-E., Cookson M.R., Civiero L. (2019). Glial Phagocytic Clearance in Parkinson’s Disease. Mol. Neurodegener..

[111] Lee J.-H., Kim J.-Y., Noh S., Lee H., Lee S.Y., Mun J.Y., Park H., Chung W.-S. (2021). Astrocytes Phagocytose Adult Hippocampal Synapses for Circuit Homeostasis. Nature.

[112] Morales I., Sanchez A., Puertas-Avendaño R., Rodriguez-Sabate C., Perez-Barreto A., Rodriguez M. (2020). Neuroglial Transmitophagy and Parkinson’s Disease. Glia.

[113] Söllvander S., Nikitidou E., Brolin R., Söderberg L., Sehlin D., Lannfelt L., Erlandsson A. (2016). Accumulation of Amyloid-β by Astrocytes Result in Enlarged Endosomes and Microvesicle-Induced Apoptosis of Neurons. Mol. Neurodegener..

[114] Beretta C., Nikitidou E., Streubel-Gallasch L., Ingelsson M., Sehlin D., Erlandsson A. (2020). Extracellular Vesicles from Amyloid-β Exposed Cell Cultures Induce Severe Dysfunction in Cortical Neurons. Sci. Rep..

[115] Tarasoff-Conway J.M., Carare R.O., Osorio R.S., Glodzik L., Butler T., Fieremans E., Axel L., Rusinek H., Nicholson C., Zlokovic B.V. (2015). Clearance Systems in the Brain-Implications for Alzheimer Disease. Nat. Rev. Neurol..

[116] Iliff J.J., Lee H., Yu M., Feng T., Logan J., Nedergaard M., Benveniste H. (2013). Brain-Wide Pathway for Waste Clearance Captured by Contrast-Enhanced MRI. J. Clin. Invest..

[117] Rasmussen M.K., Mestre H., Nedergaard M. (2018). The Glymphatic Pathway in Neurological Disorders. Lancet. Neurol..

[118] Iliff J.J., Nedergaard M. (2013). Is There a Cerebral Lymphatic System?. Stroke.

[119] Bellesi M., de Vivo L., Chini M., Gilli F., Tononi G., Cirelli C. (2017). Sleep Loss Promotes Astrocytic Phagocytosis and Microglial Activation in Mouse Cerebral Cortex. J. Neurosci. Off. J. Soc. Neurosci..

[120] Cahoy J.D., Emery B., Kaushal A., Foo L.C., Zamanian J.L., Christopherson K.S., Xing Y., Lubischer J.L., Krieg P.A., Krupenko S.A. (2008). A Transcriptome Database for Astrocytes, Neurons, and Oligodendrocytes: A New Resource for Understanding Brain Development and Function. J. Neurosci. Off. J. Soc. Neurosci..

[121] Jha M.K., Jo M., Kim J.-H., Suk K. (2019). Microglia-Astrocyte Crosstalk: An Intimate Molecular Conversation. Neuroscientist.

[122] Lagalwar S. (2022). Mechanisms of Tunneling Nanotube-Based Propagation of Neurodegenerative Disease Proteins. Front. Mol. Neurosci..

[123] Khattar K.E., Safi J., Rodriguez A.-M., Vignais M.-L. (2022). Intercellular Communication in the Brain through Tunneling Nanotubes. Cancers.

[124] Chung W.-S., Welsh C.A., Barres B.A., Stevens B. (2015). Do Glia Drive Synaptic and Cognitive Impairment in Disease?. Nat. Neurosci..

[125] Santello M., Toni N., Volterra A. (2019). Astrocyte Function from Information Processing to Cognition and Cognitive Impairment. Nat. Neurosci..

[126] Terry R.D., Masliah E., Salmon D.P., Butters N., DeTeresa R., Hill R., Hansen L.A., Katzman R. (1991). Physical Basis of Cognitive Alterations in Alzheimer’s Disease: Synapse Loss Is the Major Correlate of Cognitive Impairment. Ann Neurol.

[127] Revuelta G.J., Rosso A., Lippa C.F. (2008). Neuritic Pathology as a Correlate of Synaptic Loss in Dementia with Lewy Bodies. Am. J. Alzheimers. Dis. Other Demen..

[128] Pekny M., Pekna M., Messing A., Steinhäuser C., Lee J.-M., Parpura V., Hol E.M., Sofroniew M.V., Verkhratsky A. (2016). Astrocytes: A Central Element in Neurological Diseases. Acta Neuropathol..

[129] Booth H.D.E., Hirst W.D., Wade-Martins R. (2017). The Role of Astrocyte Dysfunction in Parkinson’s Disease Pathogenesis. Trends Neurosci..

[130] Oberheim N.A., Takano T., Han X., He W., Lin J.H., Wang F., Xu Q., Wyatt J.D., Pilcher W., Ojemann J.G. (2009). Uniquely Hominid Features of Adult Human Astrocytes. J Neurosci.

[131] Oberheim N.A., Wang X., Goldman S., Nedergaard M. (2006). Astrocytic Complexity Distinguishes the Human Brain. Trends Neurosci..

[132] Brück D., Wenning G.K., Stefanova N., Fellner L. (2016). Glia and Alpha-Synuclein in Neurodegeneration: A Complex Interaction. Neurobiol. Dis..

[133] Koob A.O., Ubhi K., Paulsson J.F., Kelly J., Rockenstein E., Mante M., Adame A., Masliah E. (2010). Lovastatin Ameliorates Alpha-Synuclein Accumulation and Oxidation in Transgenic Mouse Models of Alpha-Synucleinopathies. Exp Neurol.

[134] Dening Y., Straßl T., Ruf V., Dirscherl P., Chovsepian A., Stievenard A., Khairnar A., Schmidt F., Giesert F., Herms J. (2022). Toxicity of Extracellular Alpha-Synuclein Is Independent of Intracellular Alpha-Synuclein. Sci. Rep..

[135] Fellner L., Gabassi E., Haybaeck J., Edenhofer F. (2021). Autophagy in α-Synucleinopathies-An Overstrained System. Cells.

[136] Stefanis L., Emmanouilidou E., Pantazopoulou M., Kirik D., Vekrellis K., Tofaris G.K. (2019). How Is Alpha-Synuclein Cleared from the Cell?. J. Neurochem..

[137] Koob A.O., Sacchetti P., Singh S., Joshi N. (2019). Astroctyes and the Synucleinopathies. Pathology, Prevention and Therapeutics of Neurodegenerative Disease.

[138] Spillantini M.G., Schmidt M.L., Lee V.M.Y., Trojanowski J.Q., Jakes R., Goedert M. (1997). α-Synuclein in Lewy Bodies [8]. Nature.

[139] Van Der Putten H., Wiederhold K.H., Probst A., Barbieri S., Mistl C., Danner S., Kauffmann S., Hofele K., Spooren W.P.J.M., Ruegg M.A. (2000). Neuropathology in Mice Expressing Human α-Synuclein. J. Neurosci..

[140] Peelaerts W., Bousset L., Van der Perren A., Moskalyuk A., Pulizzi R., Giugliano M., Van den Haute C., Melki R., Baekelandt V. (2015). Alpha-Synuclein Strains Cause Distinct Synucleinopathies after Local and Systemic Administration. Nature.

[141] Lindstrom V., Gustafsson G., Sanders L.H., Howlett E.H., Sigvardson J., Kasrayan A., Ingelsson M., Bergstrom J., Erlandsson A. (2017). Extensive Uptake of Alpha-Synuclein Oligomers in Astrocytes Results in Sustained Intracellular Deposits and Mitochondrial Damage. Mol. Cell. Neurosci..

[142] Mahul-Mellier A.-L., Burtscher J., Maharjan N., Weerens L., Croisier M., Kuttler F., Leleu M., Knott G.W., Lashuel H.A. (2020). The Process of Lewy Body Formation, Rather than Simply α-Synuclein Fibrillization, Is One of the Major Drivers of Neurodegeneration. Proc. Natl. Acad. Sci. USA.

[143] Parpura V., Heneka M.T., Montana V., Oliet S.H.R., Schousboe A., Haydon P.G., Stout R.F., Spray D.C., Reichenbach A., Pannicke T. (2012). Glial Cells in (Patho)Physiology. J. Neurochem..

[144] Braidy N., Gai W.-P., Xu Y.H., Sachdev P., Guillemin G.J., Jiang X.-M., Ballard J.W.O., Horan M.P., Fang Z.M., Chong B.H. (2013). Uptake and Mitochondrial Dysfunction of Alpha-Synuclein in Human Astrocytes, Cortical Neurons and Fibroblasts. Transl. Neurodegener..

[145] Rostami J., Holmqvist S., Lindström V., Sigvardson J., Westermark G.T., Ingelsson M., Bergström J., Roybon L., Erlandsson A. (2017). Human Astrocytes Transfer Aggregated Alpha-Synuclein via Tunneling Nanotubes. J. Neurosci..

[146] Altay M.F., Liu A.K.L., Holton J.L., Parkkinen L., Lashuel H.A. (2022). Prominent Astrocytic Alpha-Synuclein Pathology with Unique Post-Translational Modification Signatures Unveiled across Lewy Body Disorders. Acta Neuropathol. Commun..

[147] di Domenico A., Carola G., Calatayud C., Pons-Espinal M., Munoz J.P., Richaud-Patin Y., Fernandez-Carasa I., Gut M., Faella A., Parameswaran J. (2019). Patient-Specific IPSC-Derived Astrocytes Contribute to Non-Cell-Autonomous Neurodegeneration in Parkinson’s Disease. Stem cell reports.

[148] Erustes A.G., Stefani F.Y., Terashima J.Y., Stilhano R.S., Monteforte P.T., da Silva Pereira G.J., Han S.W., Calgarotto A.K., Hsu Y.-T., Ureshino R.P. (2018). Overexpression of Alpha-Synuclein in an Astrocyte Cell Line Promotes Autophagy Inhibition and Apoptosis. J. Neurosci. Res..

[149] Choi I., Kim J., Jeong H.-K., Kim B., Jou I., Park S.M., Chen L., Kang U.-J., Zhuang X., Joe E.-H. (2013). PINK1 Deficiency Attenuates Astrocyte Proliferation through Mitochondrial Dysfunction, Reduced AKT and Increased P38 MAPK Activation, and Downregulation of EGFR. Glia.

[150] Pickrell A.M., Youle R.J. (2015). The Roles of PINK1, Parkin, and Mitochondrial Fidelity in Parkinson’s Disease. Neuron.

[151] Eng L.F., Ghirnikar R.S. (1994). GFAP and Astrogliosis. Brain Pathol..

[152] Sofroniew M.V., Vinters H. (2010). V Astrocytes: Biology and Pathology. Acta Neuropathol.

[153] Sofroniew M.V., Barres B.A., Freeman M.R., Stevens B. (2015). Astrogliosis. Glia.

[154] Anderson M.A., Ao Y., Sofroniew M. (2014). V Heterogeneity of Reactive Astrocytes. Neurosci. Lett..

[155] Basurco L., Abellanas M.A., Ayerra L., Conde E., Vinueza-Gavilanes R., Luquin E., Vales A., Vilas A., Martin-Uriz P.S., Tamayo I. (2022). Microglia and Astrocyte Activation Is Region-Dependent in the α-Synuclein Mouse Model of Parkinson’s Disease. Glia.

[156] Surguchev A.A., Emamzadeh F.N., Surguchov A. (2019). Cell Responses to Extracellular α-Synuclein. Molecules.

[157] Fellner L., Irschick R., Schanda K., Reindl M., Klimaschewski L., Poewe W., Wenning G.K., Stefanova N. (2013). Toll-like Receptor 4 Is Required for Alpha-Synuclein Dependent Activation of Microglia and Astroglia. Glia.

[158] Rannikko E.H., Weber S.S., Kahle P.J. (2015). Exogenous Alpha-Synuclein Induces Toll-like Receptor 4 Dependent Inflammatory Responses in Astrocytes. BMC Neurosci..

[159] Jellinger K.A. (2003). Neuropathological Spectrum of Synucleinopathies. Mov. Disord..

[160] Koob A.O., Paulino A.D., Masliah E. (2010). GFAP Reactivity, Apolipoprotein E Redistribution and Cholesterol Reduction in Human Astrocytes Treated with Alpha-Synuclein. Neurosci Lett.

[161] Vieira B.D.M., Radford R.A., Chung R.S., Guillemin G.J., Pountney D.L. (2015). Neuroinflammation in Multiple System Atrophy: Response to and Cause of Alpha-Synuclein Aggregation. Front. Cell. Neurosci..

[162] Shults C.W., Rockenstein E., Crews L., Adame A., Mante M., Larrea G., Hashimoto M., Song D., Iwatsubo T., Tsuboi K. (2005). Neurological and Neurodegenerative Alterations in a Transgenic Mouse Model Expressing Human Alpha-Synuclein under Oligodendrocyte Promoter: Implications for Multiple System Atrophy. J. Neurosci..

[163] Sofroniew M. (2009). V Molecular Dissection of Reactive Astrogliosis and Glial Scar Formation. Trends Neurosci.

[164] Singh S., Swarnkar S., Goswami P., Nath C. (2011). Astrocytes and Microglia: Responses to Neuropathological Conditions. Int. J. Neurosci..

[165] Tang G., Yue Z., Talloczy Z., Goldman J.E. (2008). Adaptive Autophagy in Alexander Disease-Affected Astrocytes. Autophagy.

[166] Şengül B., Dursun E., Verkhratsky A., Gezen-Ak D. (2021). Overexpression of α-Synuclein Reorganises Growth Factor Profile of Human Astrocytes. Mol. Neurobiol..

[167] Polymeropoulos M.H., Lavedan C., Leroy E., Ide S.E., Dehejia A., Dutra A., Pike B., Root H., Rubenstein J., Boyer R. (1997). Mutation in the α-Synuclein Gene Identified in Families with Parkinson’s Disease. Science.

[168] Goedert M. (2015). NEURODEGENERATION. Alzheimer’s and Parkinson’s Diseases: The Prion Concept in Relation to Assembled Abeta, Tau, and Alpha-Synuclein. Science.

[169] Sacino A.N., Brooks M., Thomas M.A., McKinney A.B., McGarvey N.H., Rutherford N.J., Ceballos-Diaz C., Robertson J., Golde T.E., Giasson B.I. (2014). Amyloidogenic Alpha-Synuclein Seeds Do Not Invariably Induce Rapid, Widespread Pathology in Mice. Acta Neuropathol..

[170] Prusiner S.B., Woerman A.L., Mordes D.A., Watts J.C., Rampersaud R., Berry D.B., Patel S., Oehler A., Lowe J.K., Kravitz S.N. (2015). Evidence for Alpha-Synuclein Prions Causing Multiple System Atrophy in Humans with Parkinsonism. Proc. Natl. Acad. Sci. USA.

[171] Barba L., Abu Rumeileh S., Bellomo G., Paolini Paoletti F., Halbgebauer S., Oeckl P., Steinacker P., Massa F., Gaetani L., Parnetti L. (2023). Cerebrospinal Fluid β-Synuclein as a Synaptic Biomarker for Preclinical Alzheimer’s Disease. J. Neurol. Neurosurg. Psychiatry.

[172] Bertoncini C.W., Rasia R.M., Lamberto G.R., Binolfi A., Zweckstetter M., Griesinger C., Fernandez C.O. (2007). Structural Characterization of the Intrinsically Unfolded Protein Beta-Synuclein, a Natural Negative Regulator of Alpha-Synuclein Aggregation. J. Mol. Biol..

[173] Williams J.K., Yang X., Baum J. (2018). Interactions between the Intrinsically Disordered Proteins β-Synuclein and α-Synuclein. Proteomics.

[174] Nakajo S., Shioda S., Nakai Y., Nakaya K. (1994). Localization of Phosphoneuroprotein 14 (PNP 14) and Its MRNA Expression in Rat Brain Determined by Immunocytochemistry and in Situ Hybridization. Brain Res. Mol. Brain Res..

[175] Williams J.K., Yang X., Atieh T.B., Olson M.P., Khare S.D., Baum J. (2018). Multi-Pronged Interactions Underlie Inhibition of α-Synuclein Aggregation by β-Synuclein. J. Mol. Biol..

[176] Janowska M.K., Wu K.-P., Baum J. (2015). Unveiling Transient Protein-Protein Interactions That Modulate Inhibition of Alpha-Synuclein Aggregation by Beta-Synuclein, a Pre-Synaptic Protein That Co-Localizes with Alpha-Synuclein. Sci. Rep..

[177] Yang X., Williams J.K., Yan R., Mouradian M.M., Baum J. (2019). Increased Dynamics of α-Synuclein Fibrils by β-Synuclein Leads to Reduced Seeding and Cytotoxicity. Sci. Rep..

[178] Ninkina N., Millership S.J., Peters O.M., Connor-Robson N., Chaprov K., Kopylov A.T., Montoya A., Kramer H., Withers D.J., Buchman V.L. (2021). β-Synuclein Potentiates Synaptic Vesicle Dopamine Uptake and Rescues Dopaminergic Neurons from MPTP-Induced Death in the Absence of Other Synucleins. J. Biol. Chem..

[179] Petrelli F., Dallérac G., Pucci L., Calì C., Zehnder T., Sultan S., Lecca S., Chicca A., Ivanov A., Asensio C.S. (2020). Dysfunction of Homeostatic Control of Dopamine by Astrocytes in the Developing Prefrontal Cortex Leads to Cognitive Impairments. Mol. Psychiatry.

[180] da Costa C.A., Masliah E., Checler F. (2003). Beta-Synuclein Displays an Antiapoptotic P53-Dependent Phenotype and Protects Neurons from 6-Hydroxydopamine-Induced Caspase 3 Activation: Cross-Talk with Alpha-Synuclein and Implication for Parkinson’s Disease. J. Biol. Chem..

[181] Hayashi J., Carver J.A. (2022). β-Synuclein: An Enigmatic Protein with Diverse Functionality. Biomolecules.

[182] Leitao A., Bhumkar A., Hunter D.J.B., Gambin Y., Sierecki E. (2018). Unveiling a Selective Mechanism for the Inhibition of α-Synuclein Aggregation by β-Synuclein. Int. J. Mol. Sci..

[183] Brown J.W.P., Buell A.K., Michaels T.C.T., Meisl G., Carozza J., Flagmeier P., Vendruscolo M., Knowles T.P.J., Dobson C.M., Galvagnion C. (2016). Beta-Synuclein Suppresses Both the Initiation and Amplification Steps of Alpha-Synuclein Aggregation via Competitive Binding to Surfaces. Sci. Rep..

[184] Taschenberger G., Toloe J., Tereshchenko J., Akerboom J., Wales P., Benz R., Becker S., Outeiro T.F., Looger L.L., Bähr M. (2013). β-Synuclein Aggregates and Induces Neurodegeneration in Dopaminergic Neurons. Ann. Neurol..

[185] Lodygin D., Hermann M., Schweingruber N., Flügel-Koch C., Watanabe T., Schlosser C., Merlini A., Körner H., Chang H.-F., Fischer H.J. (2019). β-Synuclein-Reactive T Cells Induce Autoimmune CNS Grey Matter Degeneration. Nature.

[186] Wei J., Fujita M., Nakai M., Waragai M., Watabe K., Akatsu H., Rockenstein E., Masliah E., Hashimoto M. (2007). Enhanced Lysosomal Pathology Caused by Beta-Synuclein Mutants Linked to Dementia with Lewy Bodies. J. Biol. Chem..

[187] Ohtake H., Limprasert P., Fan Y., Onodera O., Kakita A., Takahashi H., Bonner L.T., Tsuang D.W., Murray I.V.J., Lee V.M.-Y. (2004). Beta-Synuclein Gene Alterations in Dementia with Lewy Bodies. Neurology.

[188] Sekiyama K., Fujita M., Sekigawa A., Takamatsu Y., Waragai M., Takenouchi T., Sugama S., Hashimoto M. (2012). Ibuprofen Ameliorates Protein Aggregation and Astrocytic Gliosis, but Not Cognitive Dysfunction, in a Transgenic Mouse Expressing Dementia with Lewy Bodies-Linked P123H Beta-Synuclein. Neurosci. Lett..

[189] Hagihara H., Fujita M., Umemori J., Hashimoto M., Miyakawa T. (2018). Immature-like Molecular Expression Patterns in the Hippocampus of a Mouse Model of Dementia with Lewy Body-Linked Mutant β-Synuclein. Mol. Brain.

[190] Fujita M., Sugama S., Sekiyama K., Sekigawa A., Tsukui T., Nakai M., Waragai M., Takenouchi T., Takamatsu Y., Wei J. (2010). A β-Synuclein Mutation Linked to Dementia Produces Neurodegeneration When Expressed in Mouse Brain. Nat. Commun..

[191] Barba L., Paolini Paoletti F., Bellomo G., Gaetani L., Halbgebauer S., Oeckl P., Otto M., Parnetti L. (2022). Alpha and Beta Synucleins: From Pathophysiology to Clinical Application as Biomarkers. Mov. Disord..

[192] Psol M., Darvas S.G., Leite K., Mahajani S.U., Bähr M., Kügler S. (2021). Dementia with Lewy Bodies-Associated ß-Synuclein Mutations V70M and P123H Cause Mutation-Specific Neuropathological Lesions. Hum. Mol. Genet..

[193] Liu H., Liu W., Wu Y., Zhou Y., Xue R., Luo C., Wang L., Zhao W., Jiang J.-D., Liu J. (2005). Loss of Epigenetic Control of Synuclein-g g Gene as a Molecular Indicator of Metastasis in a Wide Range of Human Cancers. Cancer Res.

[194] Tian L., Zhao Y., Truong M.J., Lagadec C., Bourette R.P. (2018). Synuclein Gamma Expression Enhances Radiation Resistance of Breast Cancer Cells. Oncotarget.

[195] Wu K., Quan Z., Weng Z., Li F., Zhang Y., Yao X., Chen Y., Budman D., Goldberg I.D., Shi Y.E. (2007). Expression of Neuronal Protein Synuclein Gamma Gene as a Novel Marker for Breast Cancer Prognosis. Breast Cancer Res. Treat..

[196] Winham C.L., Le T., Jellison E.R., Silver A.C., Levesque A.A., Koob A.O. (2019). γ-Synuclein Induces Human Cortical Astrocyte Proliferation and Subsequent BDNF Expression and Release. Neuroscience.

[197] Le T., Winham C.L., Andromidas F., Silver A.C., Jellison E.R., Levesque A.A., Koob A.O. (2020). Chimera RNA Interference Knockdown of γ-Synuclein in Human Cortical Astrocytes Results in Mitotic Catastrophe. Neural Regen. Res..

[198] Ninkina N., Peters O., Millership S., Salem H., van der Putten H., Buchman V.L. (2009). Gamma-Synucleinopathy: Neurodegeneration Associated with Overexpression of the Mouse Protein. Hum Mol Genet.

[199] Surguchov A., Palazzo R.E., Surgucheva I. (2001). Gamma Synuclein: Subcellular Localization in Neuronal and Non-Neuronal Cells and Effect on Signal Transduction. Cell Motil. Cytoskelet..

[200] Robertson D.C., Schmidt O., Ninkina N., Jones P.A., Sharkey J., Buchman V.L. (2004). Developmental Loss and Resistance to MPTP Toxicity of Dopaminergic Neurones in Substantia Nigra Pars Compacta of Gamma-Synuclein, Alpha-Synuclein and Double Alpha/Gamma-Synuclein Null Mutant Mice. J. Neurochem..

[201] Bonda D.J., Bajić V.P., Spremo-Potparevic B., Casadesus G., Zhu X., Smith M.A., Lee H.-G. (2010). Review: Cell Cycle Aberrations and Neurodegeneration. Neuropathol. Appl. Neurobiol..

[202] Goedert M. (2001). Alpha-Synuclein and Neurodegenerative Diseases. Nat. Rev. Neurosci..

[203] Moh C., Kubiak J.Z., Bajic V.P., Zhu X., Smith M.A., Lee H.G. (2011). Cell Cycle Deregulation in the Neurons of Alzheimer’s Disease. Results Probl Cell Differ.

[204] Mohn T.C., Koob A.O. (2015). Adult Astrogenesis and the Etiology of Cortical Neurodegeneration. J. Exp. Neurosci..

[205] Andromidas F., Atashpanjeh S., Myers A.J., MacKinnon B.E., Shaffer M.M., Koob A.O. (2021). The Astrogenic Balance in the Aging Brain. Curr. Neuropharmacol..

[206] Gupta A., Inaba S., Wong O.K., Fang G., Liu J. (2003). Breast Cancer-Specific Gene 1 Interacts with the Mitotic Checkpoint Kinase BubR1. Oncogene.

[207] Pan Z.-Z.Z., Bruening W., Giasson B.I., Lee V.M.-Y.Y., Godwin A.K. (2002). γ-Synuclein Promotes Cancer Cell Survival and Inhibits Stress- and Chemotherapy Drug-Induced Apoptosis by Modulating MAPK Pathways. J. Biol. Chem..

[208] Liu Y., Tapia M.L., Yeh J., He R.C., Pomerleu D., Lee R.K. (2019). Differential Gamma-Synuclein Expression in Acute and Chronic Retinal Ganglion Cell Death in the Retina and Optic Nerve. Mol. Neurobiol..

[209] Galvin J.E., Uryu K., Lee V.M.Y., Trojanowski J.Q. (1999). Axon Pathology in Parkinson’s Disease and Lewy Body Dementia Hippocampus Contains α-, β-, and γ-Synuclein. Proc. Natl. Acad. Sci. USA.

[210] Surgucheva I., Sharov V.S., Surguchov A. (2012). γ-Synuclein: Seeding of α-Synuclein Aggregation and Transmission between Cells. Biochemistry.

[211] Edmonson C., Ziats M.N., Rennert O.M. (2014). Altered Glial Marker Expression in Autistic Post-Mortem Prefrontal Cortex and Cerebellum. Mol. Autism.

[212] Allen M., Huang B.S., Notaras M.J., Lodhi A., Barrio-Alonso E., Lituma P.J., Wolujewicz P., Witztum J., Longo F., Chen M. (2022). Astrocytes Derived from ASD Individuals Alter Behavior and Destabilize Neuronal Activity through Aberrant Ca(2+) Signaling. Mol. Psychiatry.

[213] Al-Mazidi S., Al-Ayadhi L.Y. (2021). Plasma Levels of Alpha and Gamma Synucleins in Autism Spectrum Disorder: An Indicator of Severity. Med. Princ. Pract..

[214] Masliah E., Mallory M., Hansen L., DeTeresa R., Terry R.D. (1993). Quantitative Synaptic Alterations in the Human Neocortex during Normal Aging. Neurology.

